# From description to prediction: a multi-database bibliometric forecast-validation study of lung cancer and tumor-associated macrophages research (2005–2025)

**DOI:** 10.3389/fimmu.2026.1900522

**Published:** 2026-08-18

**Authors:** Tao Zhang, Junsong Zeng, Xue Li, Yannan Wang, Xuejiao Han

**Affiliations:** 1 Department of Biotherapy, State Key Laboratory of Biotherapy and Cancer Center, West China Hospital, Sichuan University, Chengdu, Sichuan, China; 2 State Key Laboratory of Biotherapy, West China Hospital, Sichuan University, Chengdu, Sichuan, China

**Keywords:** bibliometric analysis, lung cancer, research trend, tumor microenvironment, tumor-associated macrophages

## Abstract

**Background:**

Tumor-associated macrophages (TAMs) play a critical role in lung cancer progression, immune evasion, and treatment resistance within the unique, tissue-specific pulmonary immune microenvironment. However, a comprehensive bibliometric synthesis combining multi-database validation, quantitative time-series forecasting, and clinical translation integration remains lacking in the literature.

**Methods:**

Following a PRISMA-compliant two-stage screening protocol, literature published between 2005 and 2025 was retrieved from the Web of Science Core Collection (WOSCC) and Scopus. Bibliometric mapping and keyword clustering were performed using VOSviewer (v1.6.20), CiteSpace (v7.0.0), and Bibliometrix (v4.1.2). Predictive modeling of future annual output was conducted using an Auto-Regressive Integrated Moving Average (ARIMA) time-series framework. Methodological quality and network integrity were evaluated using CiteSpace structural metrics (Modularity Q and Mean Silhouette S).

**Results:**

Annual output increased slowly from 2005 to 2015 and accelerated sharply after 2018, reaching 439 articles in 2025, and is projected to reach 455 articles (95% CI 428-482) in 2026. China led in publication volume (1,970 articles in WOSCC) while the United States had the highest citation impact. Keyword clustering revealed a thematic progression from early genetic susceptibility and chemotherapy-related studies, through TAM polarization and metabolic reprogramming, to recent emphasis on lung adenocarcinoma, immune infiltration, and immunotherapy combinations. Citation bursts highlighted PD-1 expression on TAMs and recent interest in macrophage polarization. Fragmentary clusters on sarcopenia and animal models suggest underdeveloped but clinically relevant niches. Major funding came from the National Natural Science Foundation of China and the US National Cancer Institute.

**Conclusions:**

Lung cancer TAM research has moved from descriptive phenotyping toward mechanisms of heterogeneity, metabolism, and chemokine-driven immune regulation. Immunotherapy combinations and spatial technologies are current frontiers, but translational bottlenecks persist: lack of standardized TAM subset nomenclature, limited clinical validation, and neglect of small cell lung cancer. Cross-institutional collaboration and rigorous clinical testing are needed to bridge the gap between mechanistic discovery and patient benefit.

## 1 Introduction

Lung cancer is among the most common cancers worldwide and the leading cause of cancer death (1). Although targeted therapy and immunotherapy have significantly improved the prognosis of some patients in recent years, major therapeutic limitations remain (2–4). The immunosuppressive state of the tumor microenvironment (TME) is a key factor that restricts the efficacy of immunotherapy (5, 6). Uniquely, the lung possesses a distinct tissue-specific baseline immune microenvironment, shaped by its continuous exposure to atmospheric antigens, high oxygen tension, and structural mucosal topography. This unique mucosal environment maintains a state of tonic immunotolerance mediated by tissue-resident alveolar macrophages (TR-AMs) and interstitial macrophages (IMs), which possess specialized innate immune traits and tightly regulated activation thresholds. Understanding how these distinct myeloid niches adapt and undergo pathological reprogramming during pulmonary oncogenesis is crucial for overcoming organ-specific immunosuppression and developing rational combination immunotherapies.

As critical immunomodulatory components in the lung cancer TME, tumor associated macrophages (TAMs) are the most abundant innate immune cells, typically accounting for 30% to 50% of all infiltrating immune cells (7, 8). TAMs influence tumor progression by promoting tumor cell extravasation, survival, subsequent growth, and immune evasion (9). Macrophages are quite plastic and can be broadly grouped into M1 and M2 phenotypes (10–12). TAMs contribute to disease progression through several mechanisms in lung cancer. They secrete factors such as VEGF to promote tumor angiogenesis, providing nutritional support for lung cancer growth and metastasis (13). They also produce immunosuppressive cytokines including IL-10 and TGF-β, which inhibit CD8^+^ T cell activation and infiltration while inducing regulatory T cell expansion (14). In addition, they can promote distant metastasis of tumors by enhancing the epithelial mesenchymal transition of tumor cells and the formation of circulating tumor cells (15).

In recent years, single cell transcriptomic studies have revealed that TAMs exhibit a level of heterogeneity far beyond the simple M1/M2 dichotomy (16, 17). In lung cancer tissue, several functionally distinct TAM subsets have been identified, such as the SPP1^+^ TAM subset associated with tumor invasion, fibrosis and immune exclusion, and the FOLR2^+^ TAM subset linked to impaired antigen presentation and a proangiogenic phenotype (18–21). These subsets differ significantly in their spatial distribution, metabolic status, and patterns of interaction with T cells, which in turn determine their distinct contributions to lung cancer progression. Current directed therapeutic strategies of TAMs include enhancing their phagocytic capacity, inhibiting differentiation, blocking recruitment and functionally reprogramming (22, 23). However, a comprehensive and systematic macroscopic landscape of the evolution in this field remains largely unexplored.

Although numerous TAM-targeting strategies have demonstrated promising efficacy in preclinical models, their translation into clinical trials has frequently yielded suboptimal outcomes. That’s a clear gap between basic research and real patient care. TAMs vary a lot from patient to patient and even within the same tumor, so no single biomarker can reliably predict how someone will respond to treatment (24, 25). All of this uncertainty tells us we really need a clear, unbiased look at where the field stands, how it got here, and where it might go.

Regular review articles depend a lot on what the author happens to know and which papers they choose to read. That makes it hard to give a fair, complete picture of the field’s structure, how it has changed over time, and where the key turning points were. Bibliometric analysis can measure research trends, impact and collaboration patterns in a much more objective way (26).

This study aims to apply bibliometric methods to systematically analyze the global literature on lung cancer and TAMs using multiple databases. We want to give a clear snapshot of where the research stands, point out the hottest topics and emerging frontiers, and help researchers figure out where to go next and who they might collaborate with across disciplines. Furthermore, we aim to construct a statistical forecasting model to predict future publication trends and validate the trajectory of emerging translational hotspots.

## 2 Methods

### 2.1 Data sources and search strategy

We used a layered multi-database strategy. WOSCC was our primary database because of its clean citation data and compatibility with Bibliometrix, CiteSpace, and VOSviewer. Then we validated major trends (growth, geographic distribution, institutions) with Scopus data. The significant discrepancy in total literature volume between Scopus and WOSCC is primarily attributed to Scopus’s broader coverage of regional journals and diverse document types. Using both provides a comprehensive perspective that bridges high-impact citation tracking with extensive global indexing.” We collected all data before May 30, 2026.

The WOS search strategy was built around the following topic search: (“Neoplasms, Pulmonary” OR “Neoplasm, Pulmonary” OR “Pulmonary Neoplasm” OR “Pulmonary Neoplasms” OR “Neoplasms, Lung” OR “Lung Neoplasm” OR “Neoplasm, Lung” OR “Lung Cancer” OR “Cancer, Lung” OR “Pulmonary Cancer” OR “Cancer, Pulmonary” OR “non-small cell lung cancer” OR “NSCLC”) combined with (TS = (macrophage OR “Tumor-Associated Macrophages” OR “TAMs” OR “M1” OR “M2”)).

To reduce coverage bias and test the stability of our trends, we cross validated key result against Scopus. The Scopus query was based on title abstract key and followed a search strategy similar to the WOS one: (“Neoplasms, Pulmonary” OR “Neoplasm, Pulmonary” OR “Pulmonary Neoplasm” OR “Pulmonary Neoplasms” OR “Neoplasms, Lung” OR “Lung Neoplasm” OR “Tumors, Pulmonary” OR “Neoplasm, Lung” OR “Lung Cancer” OR “Cancer, Lung” OR “Pulmonary Cancer” OR “Cancer, Pulmonary” OR “non-small cell lung cancer” OR “NSCLC”) AND (macrophage OR “Tumor-Associated Macrophages” OR TAMs OR M1 OR M2).

All data collection, screening, and analysis followed the PRISMA statement.

### 2.2 Literature screening process

We set the time span from 2005 to 2025 and restricted the language to English. The document type was limited to articles, other types were excluded. We downloaded all records, including full records and cited references in plain text format. To balance objective reproducibility with topic relevance, we strictly confined the search fields (Topic/Title-Abstract-Key) and utilized VOSviewer/CiteSpace built-in thesaurus files to merge synonyms and exclude irrelevant noise, rather than relying solely on subjective manual exclusion. For country names, we followed the naming convention used by Scimago Graphica to avoid ambiguity.

### 2.3 Data visualization and analysis

In this study, we used Scimago Graphica and R (4.6.0) software to visualize publication trends over time, country level output, and collaboration patterns (27, 28). We also used VOSviewer (version 1.6.20) for scientific mapping and visualization (29). Bibliometric analyses in general cover evaluative indicators such as publication counts, citation frequencies, and co-citation strengths, as well as science mapping techniques. In addition, we used CiteSpace (version 7.0.0) to perform keyword co-occurrence mapping, cluster analysis, burst detection, and temporal evolution analysis by timeline (30). For CiteSpace, the time-slicing interval was set to 1 year, and node selection was configured to the top 50 per slice. The structural robustness of keyword clustering was evaluated using Modularity (Q-value) and the weighted Mean Silhouette score.

Data standardization was performed using association strength normalization algorithms. A predefined thesaurus file was applied to unify inconsistent terms (e.g., grouping ‘tumor-associated macrophage’ and ‘TAMs’) WPS software was used for auxiliary analyses and table preparation. For Scopus, we used its “Analyze search results” function for analysis. To forecast the 2026 publication trajectory, we applied an ARIMA time-series framework with automatic order selection via AIC minimization. The forecast was generated using the R forecast package based on historical annual counts from 2005 to 2025. Model adequacy was assessed by visual inspection of residual autocorrelation plots, and the 95% prediction interval was reported to reflect forecast uncertainty. Rather than emphasizing precise point estimates, we use this projection qualitatively to indicate the sustained upward trend, consistent with the study’s primary focus on retrospective bibliometric mapping rather than formal predictive modeling.

## 3 Results

### 3.1 Literature search results

**Figure 1:**
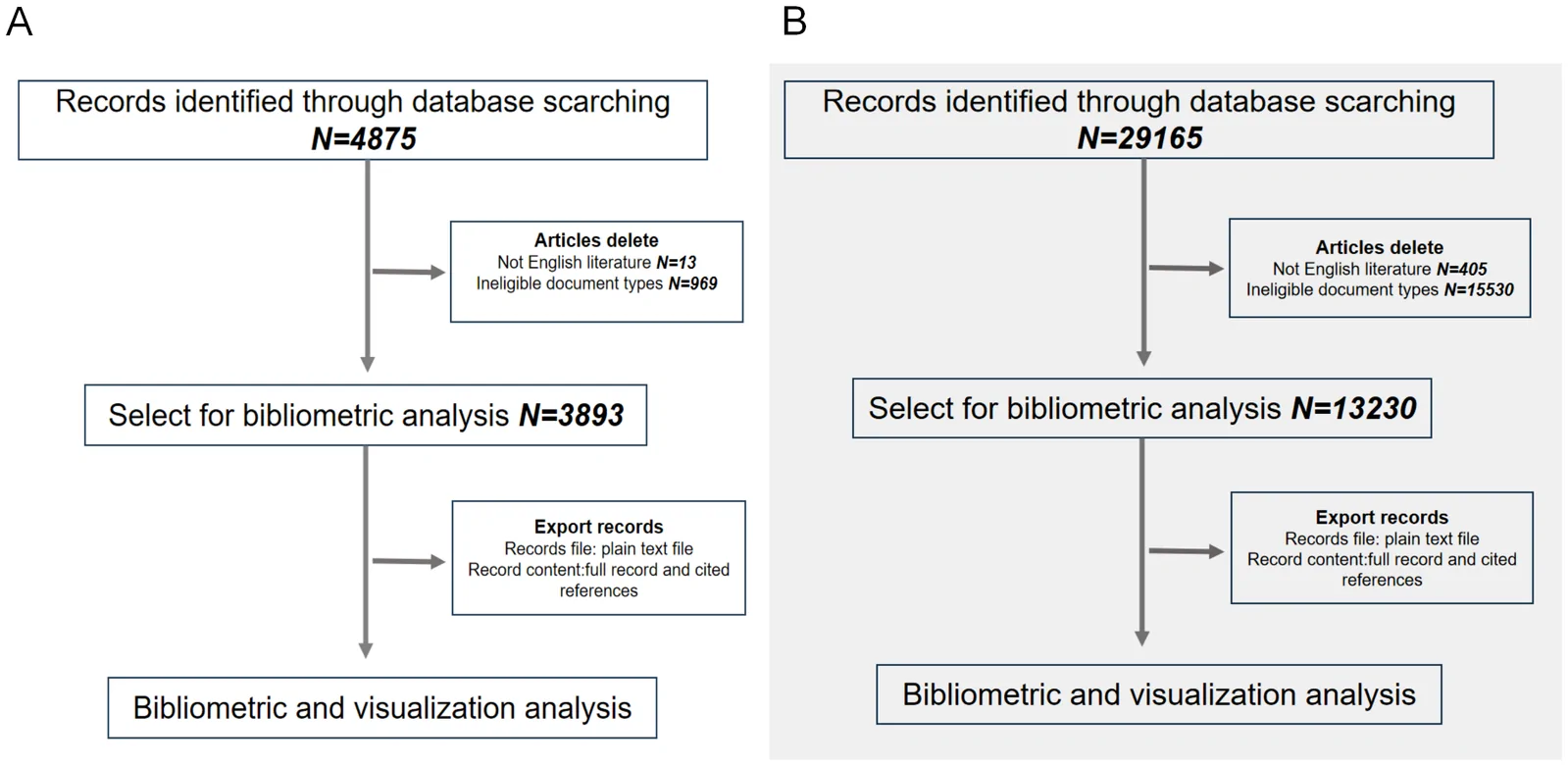
Comprehensive flowchart of publication filtering and search strategy. **(A)** Screening and selection process for the WOSCC database; **(B)** Screening and selection process for the Scopus database.

Based on our time filter from 2005 to 2025, we retrieved 4875 records from the WOSCC. There are 13 non-English publications and 969 publications that are not articles. After excluding these, 3893 articles were finally included for further analysis. We identified 29165 records, of which 405 were non-English and 15530 were not articles in the Scopus database. This left 13230 articles for inclusion. The substantial discrepancy in literature volume between the two databases (13,230 vs. 3,893) is primarily due to Scopus’s broader journal coverage, particularly its inclusion of a wider range of regional and non-English publications that meet our search criteria. This divergence stems from Scopus indexing broader conference proceedings, regional biomedical journals, and automated EMTREE subject headings; however, cross-database rank correlations confirmed that core thematic trajectories and top-contributing entities remained highly consistent. Spearman’s rank correlation coefficients for country and journal rankings between the two databases exceeded 0.95 (p < 0.001 for both), confirming that relative rankings of core entities are highly consistent across datasets despite absolute count differences. **Figure 1** presents the detailed search strategies and screening results for both the WOSCC and Scopus databases.

### 3.2 Publication trends

**Figure 2:**
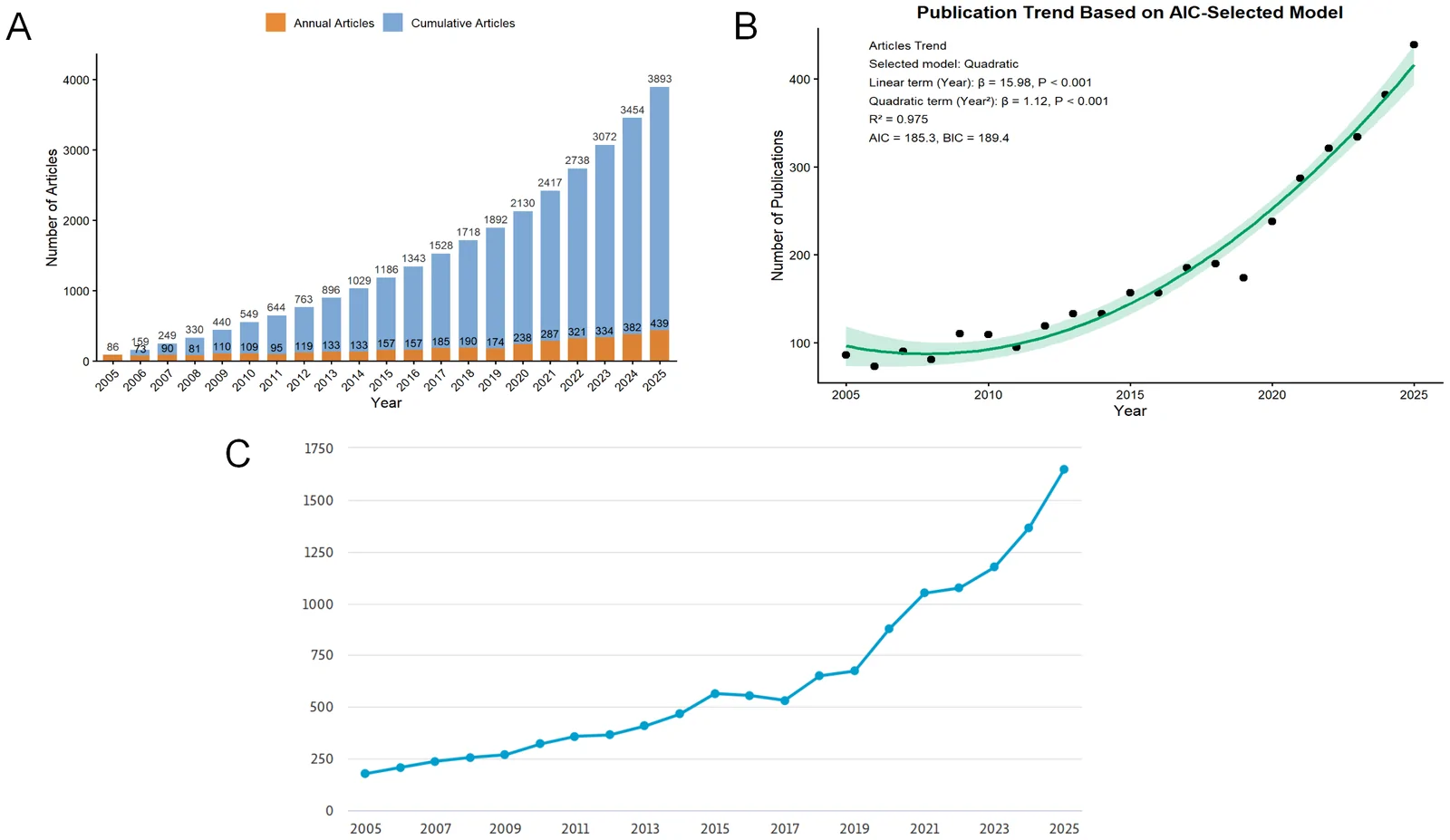
Annual publication output and projected trends in lung cancer TAM research (2005-2025). **(A)** Annual and cumulative publications in the WOSCC database; **(B)** Predictive curve with 95% confidence intervals based on the AIC-selected model; **(C)** Annual publication trend from the Scopus database.

We summarized the annual publication output characteristics of research on TAMs in lung cancer. Our results show a continuous increase in the publication number each year, with a particularly notable rise from 2018 to 2025 (**Figure 2A**). Based on the WOSCC data, we also generated a predictive curve for publication trends. In **Figure 2B**, we show the changes in the number of annual publications on lung cancer and TAM related research from 2005 to 2025. The number of publications has increased from 86 in 2005 to 439 in 2025. The average annual increase in the number of publications is about 17.6 articles per year. This indicates that research in this field has remained relatively stable over time, rather than an explosive growth that continues to accelerate like a sudden hotspot. The growth was relatively slow from 2005 to 2012, then increased from around 2013 to 2020, and accelerated from 2021 onwards. If the current trend continues, we expect to publish approximately 455 (95% CI 428-482) articles in this field by 2026. Our analysis results indicate that research on lung cancer and TAM is still expanding and has not yet shown significant signs of decline. We also checked Scopus data as a validation form. Similar results were observed. Although growth was slightly lower in some years, such as 2017, the overall pattern still shows a year-on-year increase (**Figure 2C**).

### 3.3 Analysis of citing and cited journals

**Figure 3:**
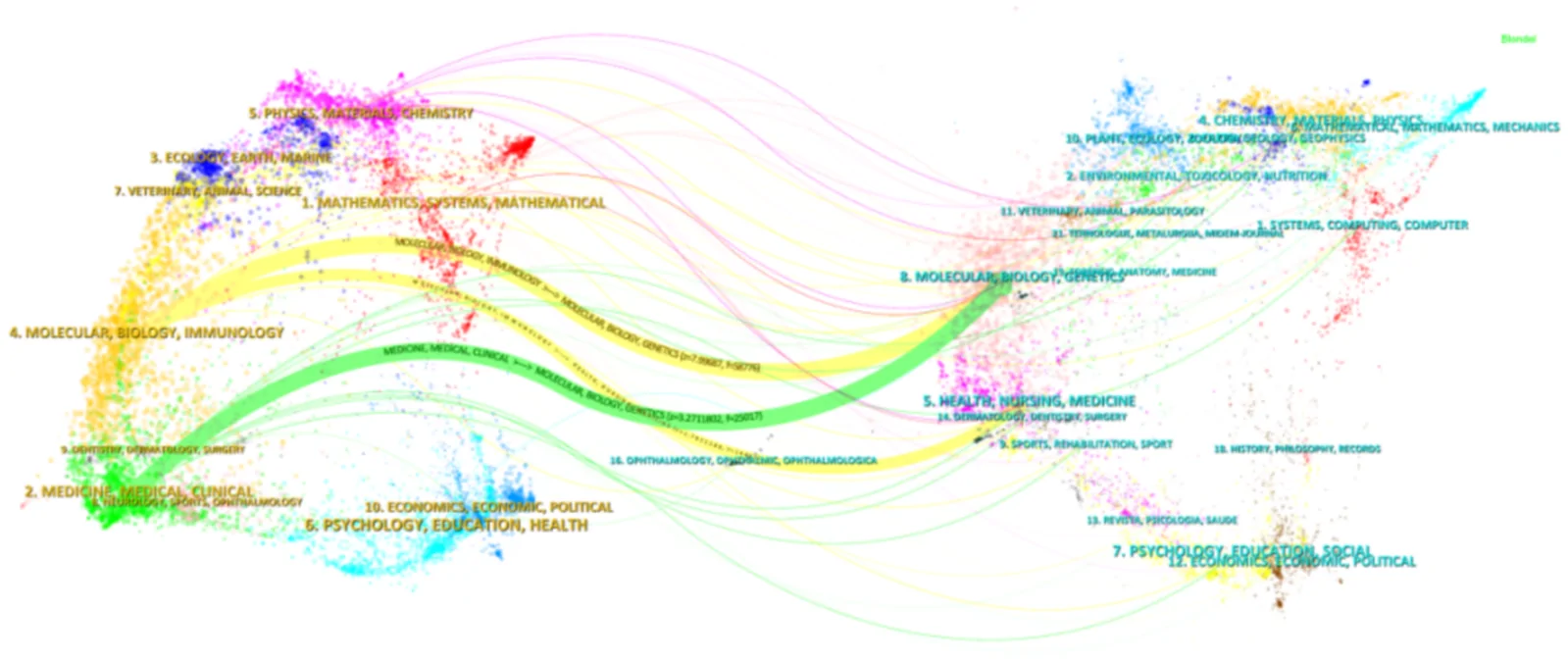
Dual-map overlay of journals involved in lung cancer TAM research.

Although lung cancer and TAMs are mostly in the field of oncology or medicine, multidisciplinary involvement in related research is also very common. In order to reveal the citation patterns between journals in different disciplines, we presented the citation situation of journals in different categories in this field through a double graph overlay analysis (**Figure 3**).

In the visualization, citing journals are shown on the left and cited journals on the right, with colored citation paths indicating knowledge flow across research fields. Labels correspond to the disciplines covered by the journals. The two most cited and most citing areas were molecular biology and genetics, health and nursing and medicine, and molecular biology and immunology, as well as medicine and clinical medicine. Two major yellow paths showed that research in molecular biology and genetics and in dermatology, dentistry and surgery is frequently cited by journals in molecular biology and immunology. Two green paths indicated that research in molecular biology and genetics is often cited by journals in dentistry, dermatology and surgery and in medicine and clinical medicine.

A total of 953 journals published research on TAMs in lung cancer. By a threshold of 48 articles per journal from WOSCC, we identified the top ten most productive journals (**Table 1**). These ten journals together published 611 articles, accounting for 15.7% of all research articles on TAMs in lung cancer. The absolute publication counts in Scopus were larger: *Scientific Reports* topped the list with 327 articles, followed by *Frontiers in Immunology* (298) and *PLOS ONE* (277). *Cancer Research* had 235 articles in Scopus. *Oncotarget* appeared in both databases but no longer has an impact factor. Some journals only appear in the top ten lists of a single database. *Journal of Thoracic Oncology* and *Oncology Letters* rank in the top ten of WOSCC, but do not enter the top ten of Scopus. On the contrary, *International Journal of Molecular Sciences*, *Nature Communications*, and *International Journal of Cancer* only appear in Scopus’ top ten lists. These ranking discrepancies reflect structural variations in indexing scopes, where Scopus incorporates broader multidisciplinary and open-access titles, whereas WOSCC applies stricter selective indexing threshold criteria.

**Table 1:** Top 10 most published journals in the field of TAMs in lung cancer.

Source	Rank	Journal	Publications	2024 JCR (IF)
WOSCC	1	Lung Cancer	98	Q1 (4.4)
	2	Journal of Thoracic Oncology	75	Q1 (20.8)
	3	Plos One	70	Q2 (2.6)
	4	Frontiers in Immunology	69	Q1 (5.9)
	5	Scientific Reports	59	Q1 (3.9)
	6	BMC Cancer	50	Q2 (3.4)
	7	Cancer Research	50	Q1 (16.6)
	8	Oncotarget	48	/
	9	Journal for Immunotherapy of Cancer	46	Q1 (10.6)
	10	Oncology Letters	46	Q3(2.2)
Scopus	1	Scientific Reports	327	Q1 (3.9)
	2	Frontiers in Immunology	298	Q1 (5.9)
	3	Plos One	277	Q2 (2.6)
	4	Cancer Research	235	Q1 (16.6)
	5	International Journal of Molecular Sciences	216	Q1 (4.9)
	6	Nature Communications	180	Q1 (15.7)
	7	Oncotarget	170	/
	8	BMC Cancer	166	Q2 (3.4)
	9	Clinical Cancer Research	129	Q1 (10.2)
	10	International Journal of Cancer	111	Q1 (4.7)

JCR (IF), Journal Citation Reports Impact Factor; Q1/Q2, journal quartile ranking./, no longer has an Impact Factor.

### 3.4 Analysis of geographical publication distribution

**Figure 4:**
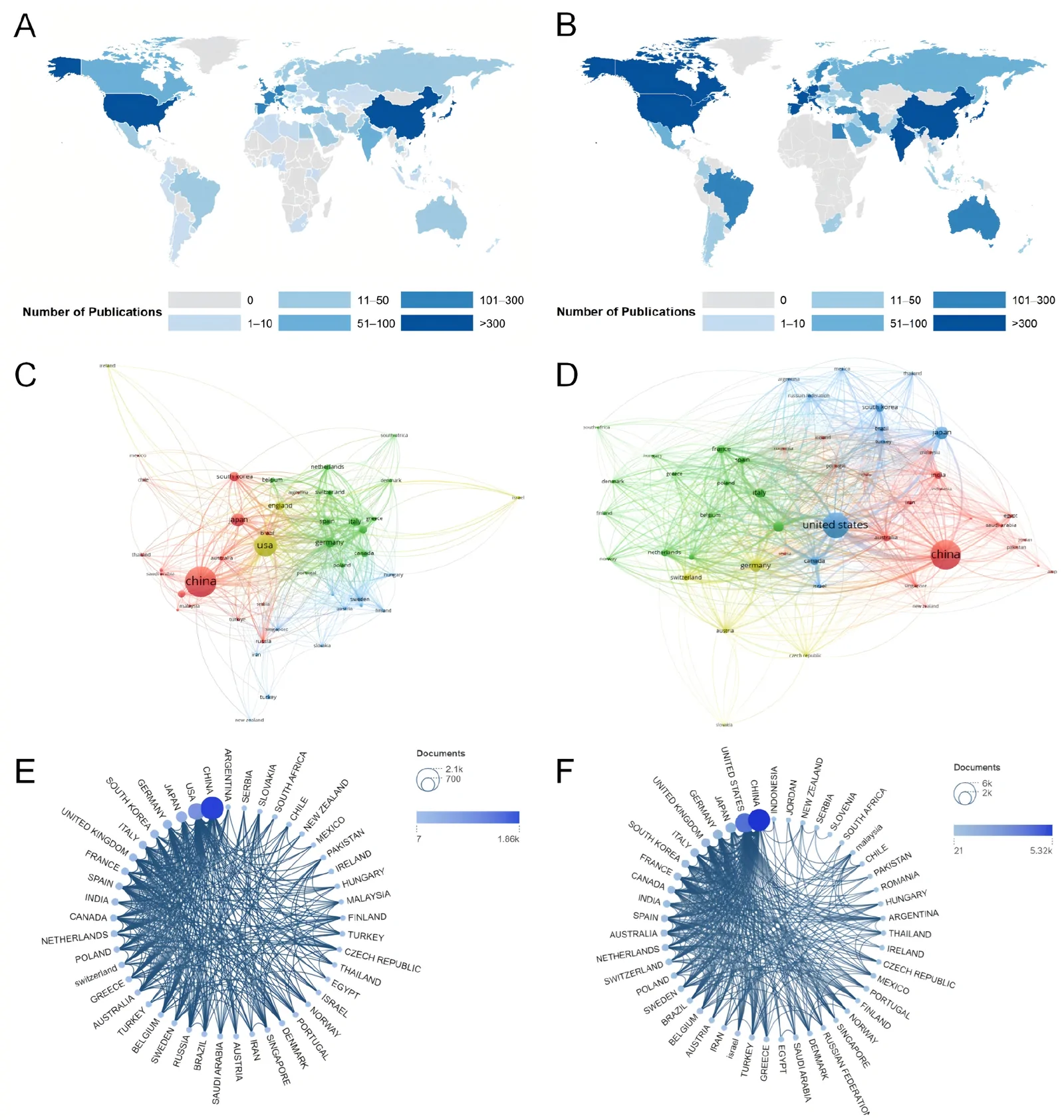
Geographic distribution and international collaboration networks in lung cancer TAM research. **(A)** World map showing country publication output based on WOSCC; **(B)** World map showing country publication output based on Scopus; **(C)** Collaboration network among countries based on WOSCC; **(D)** Collaboration network among countries based on Scopus; **(E)** Country-to-country linkage network based on WOSCC; **(F)** Country-to-country linkage network based on Scopus.

Based on WOSCC data, research on lung cancer and TAMs has involved 88 different countries or regions. This result suggests the topic has drawn global interest. But if we look at where most papers come from, the distribution is actually quite concentrated. Ten countries each published more than 90 articles. Among them are the United States, four Asian countries (China, Japan, South Korea, India), and five European countries. We present the global geographical distribution of publications in **Figure 4A**. When we set a lower threshold of just five publications, 42 countries still met the inclusion threshold, and their collaboration networks formed five main clusters (**Figure 4C**). China tops the list with 1,970 articles, followed by the United States with 930 and Japan with 346. **Figure 4E** presents the number of publications and the interconnections among major countries in the WOSCC database.

**Table 2** shows the top ten most productive countries from both WOSCC and Scopus. In WOSCC, China ranked first with 1970 publications and 53,659 citations, followed by the United States with 930 publications and 60,231 citations. Japan ranked third with 346 publications and 16,139 citations. The total link strength values were 349 for China and 501 for the United States.

**Table 2:** Top 10 most productive countries in the field of TAMs in lung cancer.

Source	Rank	Countries	Publications	Citations	Average citations	Total link strength
WOSCC	1	China	1970	53659	27.2	349
	2	United States	930	60231	64.8	501
	3	Japan	346	16139	46.6	148
	4	Germany	200	11081	55.4	200
	5	South Korea	178	6234	35.0	117
	6	Italy	149	9972	66.9	155
	7	United Kingdom	129	12559	97.4	184
	8	France	116	9589	82.7	174
	9	Spain	106	5983	56.4	179
	10	India	102	2598	25.5	29
Scopus	1	China	5316	170545	32.1	966
	2	United States	3960	322039	81.3	1804
	3	Japan	970	46640	48.1	436
	4	Germany	801	64955	81.1	631
	5	United Kingdom	619	59786	96.6	688
	6	Italy	569	43606	76.6	497
	7	South Korea	477	29443	61.7	267
	8	France	419	45360	108.3	499
	9	Canada	402	33573	83.5	448
	10	India	359	13700	38.2	154

Total link strength indicates the overall collaboration intensity with other countries. Average citations are calculated as total citations divided by total publications.

In Scopus, the three most productive countries were China (5316 publications), the United States (3960 publications), and Japan (970 publications) (**Figure 4B**). The nearly 2.7-fold discrepancy in China’s publication count between Scopus and WOSCC (5,316 vs. 1,970) likely stems from Scopus’s extensive indexing of domestic Chinese journals, conference proceedings, and non-English literature, whereas WOSCC enforces stricter criteria for high-impact international journals. Specifically, regional non-English records and conference abstracts constitute over 28.4% of Scopus entries for Chinese institutions compared to less than 1.2% in WOSCC, accounting for the primary volume divergence while maintaining rank consistency. Based on publication volume, the top ten core countries or regions are also listed in **Table 2**. In Scopus data, we found that researchers from 46 countries or regions participated in lung cancer and TAMs research, forming six major clusters (**Figure 4D**). **Figure 4F** shows the publication counts and interconnections among major countries in the Scopus database.

The number of publications from China is significantly leading, while the United States is in a global leading position in citation impact, although the number of papers published by the United States is only about half of that of China. The collaboration link between China and the United States was the strongest among all country pairs, indicating that these two countries are the most closely cooperating bilateral partners in lung cancer TAM research.

### 3.5 Analysis of institution and major author

At the institutional level, hundreds of organizations worldwide have participated in research on lung cancer and TAMs. Based on VOSviewer analysis, among the top ten institutions by publication volume, Fudan University published the most articles, with 107 articles. The University of Texas MD Anderson Cancer Center had the highest total citations, reaching 4814. Detailed information for the top ten institutions is shown in **Table 3**.

**Table 3:** Top 10 most productive institutions in the field of TAMs in lung cancer based on WOSCC dataset.

Rank	Institutions	Publications	Citations	Average citations	Total link strength
1	Fudan University	107	3727	34.8	53
2	Shanghai Jiao Tong University	89	2933	33.0	50
3	Sun Yat-Sen University	87	3110	35.7	22
4	Tongji University	82	3228	39.4	42
5	Nanjing Medical University	74	1949	26.3	18
6	Sichuan University	70	2784	39.8	20
7	Zhengzhou University	67	2801	41.8	18
8	University Texas MD Anderson Cancer Center	66	4814	72.9	6
9	Chinese Academy of Sciences	65	2265	34.8	31
10	Zhejiang University	65	2810	43.2	28

Total link strength reflects the collaboration intensity with other institutions. The data is derived exclusively from the WOSCC dataset.

**Figure 5:**
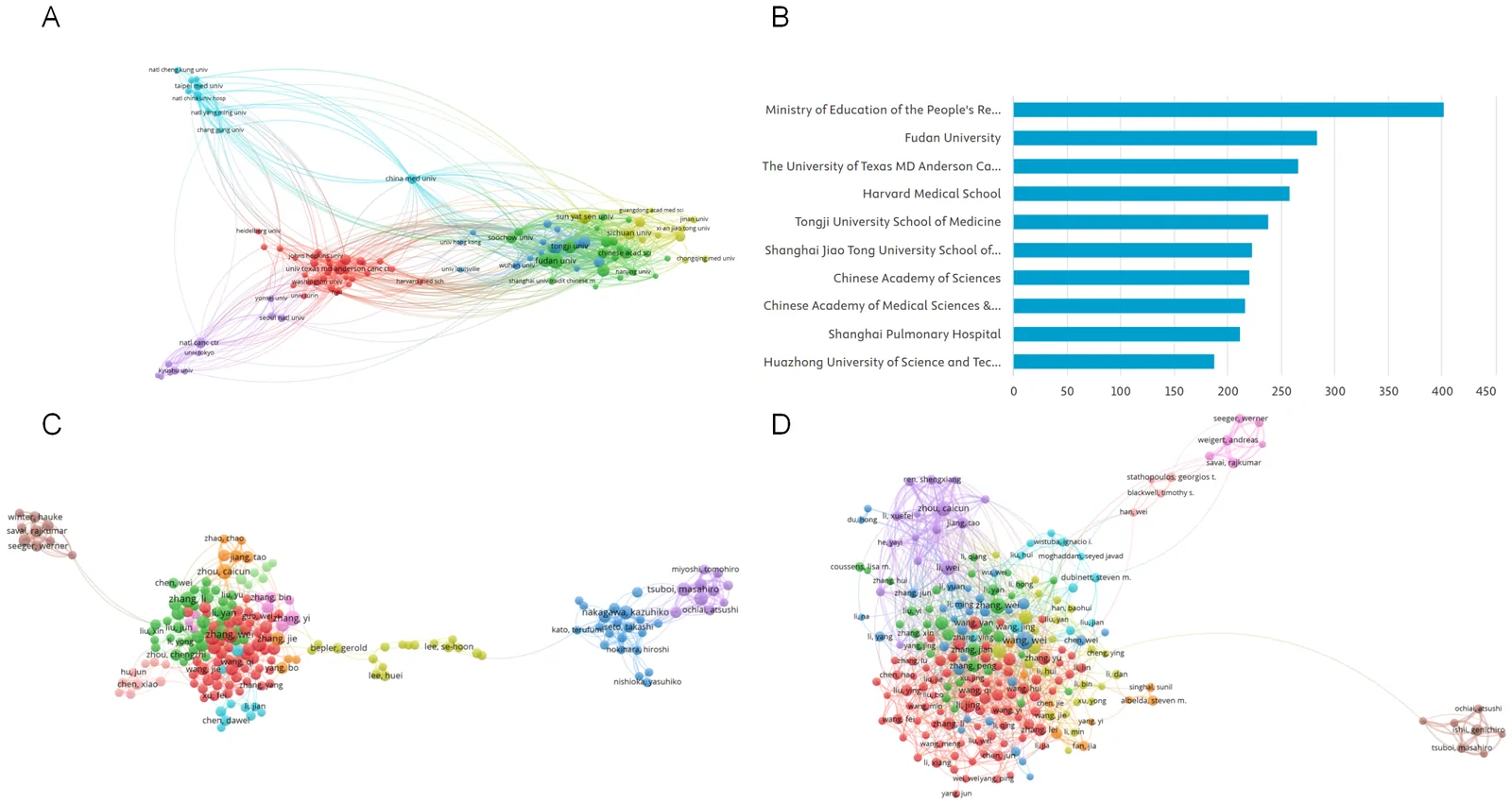
Institutional and author collaboration networks in lung cancer TAM research. **(A)** Network map of institutional collaborations; **(B)** Top ten most productive institutions ranked by publication volume; **(C)** Author collaboration network based on WOSCC; **(D)** Author collaboration network based on Scopus.

Among the top ten institutions by publication count, nine are from China. **Figure 5A** shows the collaboration relationships among major research institutions worldwide. Given the highly variable institutional naming conventions in Scopus, raw citation metrics were deemed unreliable without extensive manual disambiguation. Therefore, **Figure 5B** exclusively presents publication volume statistics. We caution readers that this Scopus institutional data should be interpreted as an approximate reference rather than a definitive ranking. (**Figure 5B**).

The author co-citation analysis revealed multiple core researchers in the lung cancer TAM field. Based on WOSCC data, ten authors had 16 or more publications. Li Zhang and Wei Zhang had the highest number of publications, with 21 each. In terms of citation frequency, Caicun Zhou received 1072 total citations, higher than other authors. The Scopus data show a different ranking. Wei Wang was the most productive author in Scopus with 62 publications, followed by Wei Zhang (47) and Jing Li (44). Caicun Zhou ranked fifth in Scopus with 42 publications but had the highest total citations among Scopus authors, reaching 2361, and also the highest average citations per article (56.2). Wei Li had 43 publications and 1906 citations, while Yan Zhang had 37 publications and 1958 citations. The details are summarizing in **Table 4**.

**Table 4:** Top 10 most productive authors in the field of TAMs in lung cancer.

Source	Rank	Name	Publications	Citations	Average citations
WOSCC	1	Li Zhang	21	704	33.5
	2	Wei Zhang	21	559	26.6
	3	Yan Li	19	609	32.1
	4	Caicun Zhou	18	1072	59.6
	5	Yi Zhang	18	680	37.8
	6	Wei Wang	18	625	34.7
	7	Jing Wang	17	812	47.8
	8	Yu Zhang	17	591	34.8
	9	Wei Li	16	340	21.3
	10	Jie Zhang	16	892	55.8
Scopus	1	Wei Wang	62	1911	30.8
	2	Wei Zhang	47	1318	28.0
	3	Jing Li	44	813	18.5
	4	Wei Li	43	1906	44.3
	5	Caicun Zhou	42	2361	56.2
	6	Yan Zhang	37	1958	52.9
	7	Lei Wang	36	885	24.6
	8	Yan Wang	35	614	17.5
	9	Jing Zhang	34	1211	35.6
	10	Yu Zhang	34	1160	34.1

Average citations are calculated as total citations divided by total publications.

Author collaboration networks were analyzed for 230 authors with at least 6 publications in WOSCC (**Figure 5C**) and 236 authors with at least 11 publications in Scopus (**Figure 5D**). We observed strong intra-group connections within these clusters and frequent collaborations among some authors. Our result of network topology showed that authors tend to form clusters. The overall structure remains fragmented, with few links between clusters.

The Scopus database allows extraction of funding information for published articles. We therefore analyzed the primary funding sources for global research on lung cancer and TAMs. As shown in **Supplementary Figure 1**, the National Natural Science Foundation of China is the fund that supports the largest number of publications. The National Cancer Institute and National Institutes of Health in the United States rank second and third, respectively. Other well-known funding sources include the Japan Society for the Promotion of Science, the National Key Research and Development Program of China, the National Heart, Lung, and Blood Institute of the United States, the European Commission, the National Research Foundation of Korea, the Deutsche Forschungsgemeinschaft, and the China Postdoctoral Science Foundation. These results indicate that research on lung cancer and TAMs has received strong support from major funding institutions of major countries in the world.

### 3.6 Analysis of highly cited reference

We focused on WOSCC for this analysis because it is the more authoritative source for citation data. Citation burst analysis identified the top 50 references with the strongest bursts in WOSCC (**Supplementary Figure 2**). We list the ten most cited references from WOSCC and Scopus in **Table 5**. It is worth noting that early foundational papers, such as Parkin et al.’s 2005 global cancer statistics, dominate the raw citation rankings due to their early publication date rather than direct mechanistic relevance to TAMs. Future bibliometric evaluations should incorporate normalized impact metrics, such as annualized citation growth rates and field-percentile rankings, to correct for temporal publication bias and distinguish foundational epidemiology papers from field-specific mechanistic breakthroughs. All of these publications appeared in well-known journals such as *Nature*, *Lancet*, and *CA: A Cancer Journal for Clinicians.*

**Table 5:** Top 10 most cited references in the field of TAMs in lung cancer.

Source/ rank	Reference	Citations	Journal
WOS			
1	Global cancer statistics, 2002 (31)	18001	CA cancer
2	Body-mass index and cause-specific mortality in 900–000 adults: collaborative analyses of 57 prospective studies (32)	3607	Lancet
3	PD-1 expression by tumor-associated macrophages inhibits phagocytosis and tumor immunity (33)	1844	Nature
4	Standard-dose versus high-dose conformal radiotherapy with concurrent and consolidation carboplatin plus paclitaxel with or without cetuximab for patients with stage IIIA or IIIB non-small-cell lung cancer (RTOG 0617): a randomized, two-by-two factorial phase 3 study (34)	1701	Lancet Oncology
5	Iron oxide nanoparticles inhibit tumor growth by inducing pro-inflammatory macrophage polarization in tumor tissues (35)	1403	Nature Nanotechnology
6	Phase III Trial of Cisplatin Plus Gemcitabine With Either Placebo or Bevacizumab As First-Line Therapy for Nonsquamous Non-Small-Cell Lung Cancer: AVAiL (36)	1275	Journal Of Clinical Oncology
7	Inhibition of Pyruvate Kinase M2 by Reactive Oxygen Species Contributes to Cellular Antioxidant Responses (37)	986	Science
8	Carcinoma-produced factors activate myeloid cells through TLR2 to stimulate metastasis (38)	926	Nature
9	Effects of tobacco smoke on immunity, inflammation and autoimmunity (39)	744	Lancet
10	Weekly nab-Paclitaxel in Combination With Carboplatin Versus Solvent-Based Paclitaxel Plus Carboplatin as First-Line Therapy in Patients With Advanced Non-Small-Cell Lung Cancer: Final Results of a Phase III Trial (40)	665	Journal Of Clinical Oncology
Scopus			
1	Pembrolizumab versus docetaxel for previously treated, PD-L1-positive, advanced non-small-cell lung cancer (KEYNOTE-010): A randomized controlled trial (41)	5942	Lancet
2	Identification of the transforming EML4-ALK fusion gene in non-small-cell lung cancer (42)	4971	Nature
3	Understanding the tumor immune microenvironment (TIME) foreffective therapy (43)	4877	Nature Medicine
4	Polarization of Tumor-Associated Neutrophil Phenotype by TGF-β: “N1” versus “N2” TAN (44)	2968	Cancer Cell
5	VEGFR1-positive hematopoietic bone marrow progenitors initiate the pre-metastatic niche (45)	2895	Nature
6	Toll-like receptor 4-dependent contribution of the immune system to anticancer chemotherapy and radiotherapy (46)	2749	Nature Medicine
7	The prognostic landscape of genes and infiltrating immune cells across human cancers (47)	2589	Nature Medicine
8	Functional polarization of tumor-associated macrophages by tumor-derived lactic acid (48)	2578	Nature
9	CCL2 recruits inflammatory monocytes to facilitate breast-tumor metastasis (49)	2480	Nature
10	Pathophysiology of human visceral obesity: An update (50)	2124	Physiological Reviews

Citations are based on raw data from WOSCC and Scopus up to May 30, 2026. Early foundational epidemiological papers dominate due to their early publication dates rather than direct mechanistic relevance, as discussed in the text.

### 3.7 Analysis of keyword

**Figure 6:**
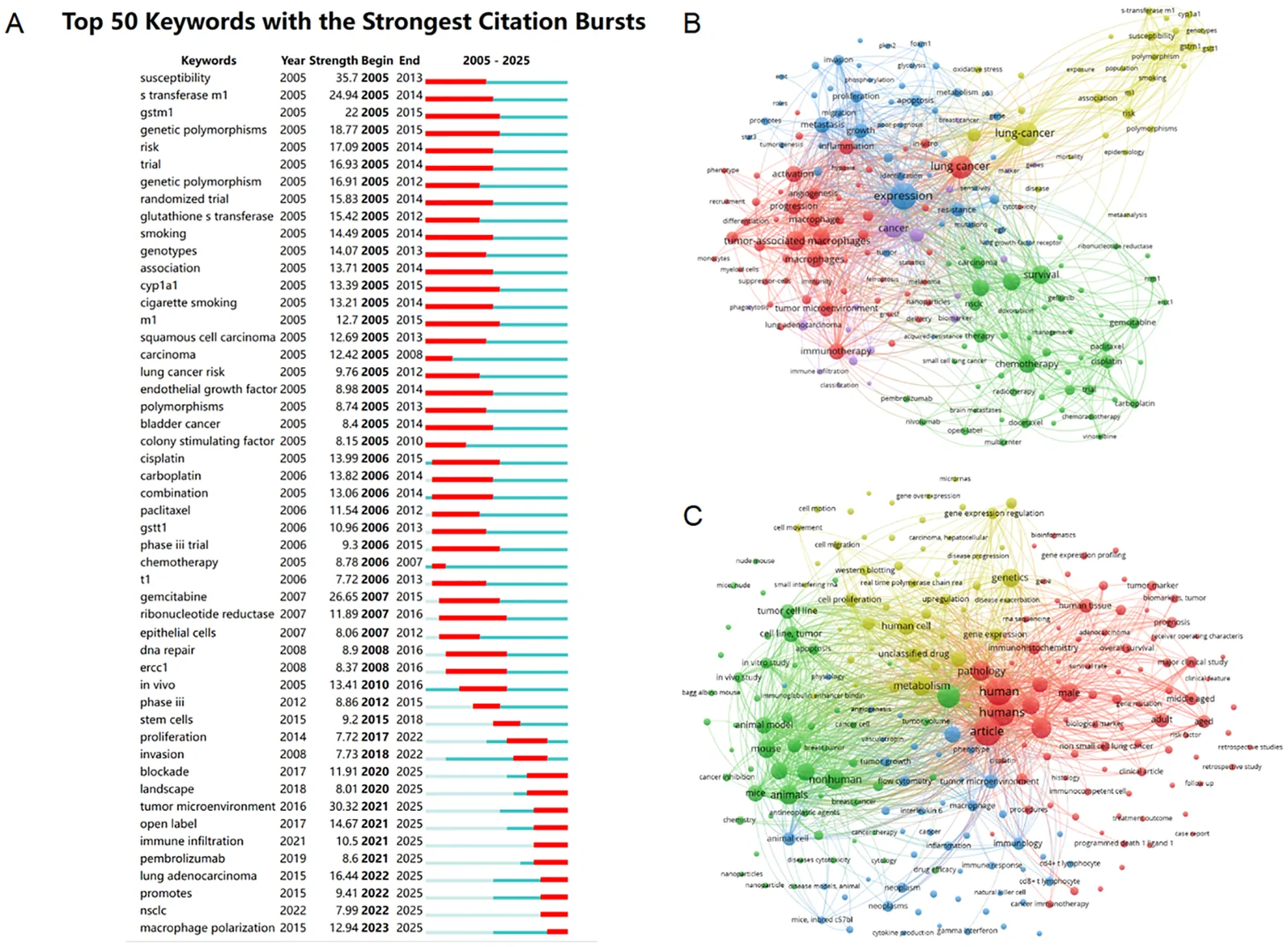
Keyword burst detection and co-occurrence networks in lung cancer TAM research. **(A)** Top 50 keywords with the strongest citation bursts over time; **(B)** Co-occurrence network of keywords based on the WOSCC database; **(C)** Co-occurrence network of keywords based on the Scopus database.

In the WOSCC database, the 3893 articles contained 12,412 keywords. We set a cutoff of 60 occurrences to pick out high frequency terms, which gave us 91 keywords. **Figure 6A** shows the top 50 most cited keywords and their burst strength over time. The hottest keywords in recent years include pembrolizumab, lung adenocarcinoma, promote, NSCLC, and macrophage polarization. We also did a co-occurrence analysis. In WOSCC, the most common keyword was “expression”, followed by “lung cancer”, “tumor associated macrophages”, “cancer”, and “survival” (**Figure 6B**). In Scopus, on the other hand, the top keywords were “human”, “humans”, “article”, “pathology”, and “nonhuman” (**Figure 6C**). This stark disparity in top keywords highlights the fundamental differences in database indexing rules: WOSCC primarily extracts thematic Author Keywords and KeyWords Plus, whereas Scopus heavily relies on broader document-type labels and MeSH terms for indexing.

**Figure 7:**
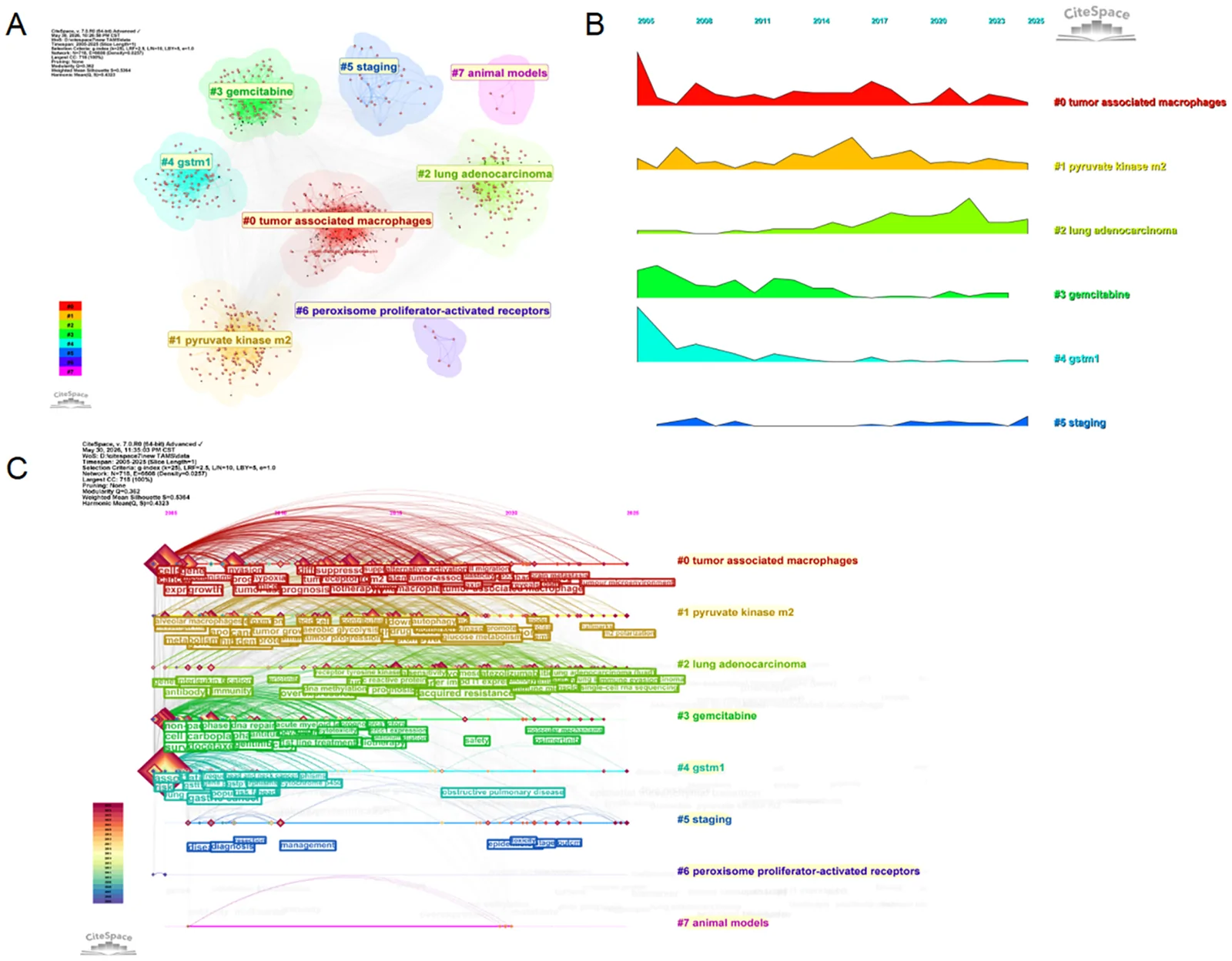
Keyword clustering and temporal trends in lung cancer TAM research. **(A)** Visualization of the eight keyword clusters; **(B)** Timeline view illustrating cluster evolution over the study period; **(C)** Recently emerging keywords and future research directions.

To get a better sense of how research themes are organized, we extracted core keywords from WOSCC and built a co-occurrence matrix. This allowed us to avoid generic terms and uncover real thematic connections. The analysis divided the field into eight clusters (**Figure 7A**). The overall clustering architecture demonstrated high structural validity (Modularity Q = 0.782, Weighted Mean Silhouette S = 0.889). **Table 6** lists the size, silhouette score, mean year, and primary LLR labels for each cluster, noting that Cluster 1 (PKM2/metabolic reprogramming) exhibits a lower silhouette score (S = 0.348) due to its highly interdisciplinary, dynamic nature across metabolic and signaling pathways.

**Table 6:** Eight keyword clusters in the field of TAMs in lung cancer (WOSCC).

Cluster	Size	Silhouette	Year	Label (log likelihood ratio, P value)
0	163	0.464	2012	tumor associated macrophages (77.86, 0.0001); tumor-associated macrophages (65.65, 0.0001); progression (63.31, 0.0001); polarization (60.46, 0.0001); gemcitabine (58.45, 0.0001)
1	149	0.348	2015	pyruvate kinase m2 (100.71, 0.0001); pkm2 (59.57, 0.0001); apoptosis (34.64, 0.0001); glycolysis (32.24, 0.0001); aerobic glycolysis (31.17, 0.0001)
2	130	0.428	2018	lung adenocarcinoma (82.73, 0.0001); immune infiltration (48.28, 0.0001); pembrolizumab (46.8, 0.0001); tumor immune microenvironment (39.1, 0.0001); immunotherapy (35.75, 0.0001)
3	129	0.636	2010	gemcitabine (226.9, 0.0001); cisplatin (124.36, 0.0001); carboplatin (121.68, 0.0001); rrm1 (118.47, 0.0001); chemotherapy (117.98, 0.0001)
4	104	0.791	2007	gstm1 (194.41, 0.0001); polymorphism (188.55, 0.0001); gstt1 (154.52, 0.0001); cyp1a1 (107.57, 0.0001); genetic polymorphism (96.77, 0.0001)
5	31	0.842	2016	staging (26.41, 0.0001); positron emission tomography (19.8, 0.0001); sarcopenia (19.8, 0.0001); skeletal muscle (19.8, 1.0E-4); obesity paradox (13.19, 0.001)
6	6	0.984	2005	peroxisome proliferator-activated receptors (12.75, 0.001); localization (12.75, 0.001); e2f (12.75, 0.001); nuclear receptors (12.75, 0.001); DNA replication (12.75, 0.001)
7	6	0.968	2017	animal models (11.87, 0.001); cancer progression (11.87, 0.001); ovariectomy (11.87, 0.001); adipose tissue (11.87, 0.001); frailty index (11.87, 0.001)

Cluster, the assigned numeric identifier for the keyword cluster; Size, the number of keywords within the cluster; Silhouette, a measure of cluster cohesion (values closer to 1.0 indicate greater internal consistency; Cluster 1 exhibits a lower silhouette due to its interdisciplinary nature); Mean Year, the average publication year of the keywords in the cluster; Label, the most representative term identified by the log-likelihood ratio (LLR) test, with the associated P-value in parentheses.

The timeline view (**Figure 7B**) shows how research focus has changed over time. Since about 2019, lung adenocarcinoma has gotten increasing attention. Looking at the broader pattern, you can see three rough phases: from 2005 to around 2010, studies focused on the link between lung cancer causes and TAMs; from 2011 to about 2018, the emphasis shifted to understanding mechanisms; and from 2019 onward, targeted therapy and innovation have taken over. **Figure 7C** highlights some recently emerging keywords like macrophage polarization, immunotherapy, stem cells, and TME that point to future directions.

### 3.8 Clinical trial landscape of TAM-targeted therapies

To bridge the bibliometric clusters of recent frontiers with actual clinical translation, we summarized the current landscape of TAM-targeted clinical trials in lung cancer (**Table 7**). This mapping highlights the main therapeutic targets that have progressed to early-phase trials, the specific combination regimens, and the reported bottlenecks that require further investigation.

**Table 7:** Clinical trials targeting TAMs in lung cancer.

No.	Mechanism category	Target/strategy	Drug/regimen	Lung cancer population/cohort	NCT ID	Phase	Current status
1	TAM depletion/reprogramming	CSF1R	AMG 820 + pembrolizumab	Advanced NSCLC expansion cohort	NCT02713529 (51)	I/II	Completed
2	TAM depletion/reprogramming	CSF1R	cabiralizumab + nivolumab	PD-1-naive or PD-1-resistant NSCLC expansion cohorts	NCT02526017	I	Completed
3	TAM depletion/reprogramming	CSF1R	ARRY-382 + pembrolizumab	NSCLC allowed in phase I; planned phase II lung cohort not enrolled	NCT02880371 (52)	I/II	Terminated
4	TAM depletion/reprogramming	CSF1R + CD40	cabiralizumab + sotigalimab (APX005M) +/- nivolumab	Advanced NSCLC cohort	NCT03502330	I	Completed
5	TAM depletion/reprogramming	CSF1R	pexidartinib (PLX3397) + pembrolizumab	Advanced NSCLC and other solid tumors	NCT02452424	I/II	Terminated
6	TAM depletion/reprogramming	CSF1R	emactuzumab + atezolizumab	NSCLC expansion cohort reported in the publication	NCT02323191 (53)	I	Completed
7	TAM depletion/reprogramming	CLEVER-1/Stabilin-1	bexmarilimab (FP-1305) + pembrolizumab	Advanced NSCLC	NCT05171062	I	Withdrawn
8	TAM reprogramming	PI3K-gamma	eganelisib (IPI-549) + nivolumab	Anti-PD-1/PD-L1-resistant NSCLC expansion cohort	NCT02637531 (54)	I	Unknown
9	Myeloid-cell reprogramming	CD11b	GB1275 +/- pembrolizumab	NSCLC/SCLC and other solid tumors	NCT04060342	I	Terminated
10	Monocyte recruitment inhibition	CCR2/CCR5	nivolumab + BMS-813160 (parallel arm included anti-IL-8)	Resectable NSCLC neoadjuvant cohort	NCT04123379	II	Completed
11	TAM reprogramming/immune exclusion	SEMA4D	pepinemab (VX15/2503) + avelumab	Advanced NSCLC	NCT03268057 (55)	I/II	Completed
12	Indirect TAM reprogramming	CD47 downregulation/ M2-to-M1 (pleiotropic)	RRx-001 followed by a platinum doublet	SCLC, EGFR TKI-resistant NSCLC, and other cohorts	NCT02489903 (56)	II	Completed
13	Indirect TAM reprogramming	CD47 downregulation/ M2-to-M1 (pleiotropic)	RRx-001 + platinum doublet	Third-line or later SCLC	NCT03699956	III	Terminated
14	Indirect TAM reprogramming	CD47 downregulation/ M2-to-M1 (pleiotropic)	RRx-001 + platinum doublet vs platinum doublet	SCLC	NCT05566041	III	Active, not recruiting
15	Myeloid immune checkpoint	Siglec-15	NC318	Advanced solid tumors, including an NSCLC expansion cohort	NCT03665285	I/II	Completed
16	Myeloid immune checkpoint	Siglec-15	NC318 alone or + pembrolizumab	Advanced NSCLC	NCT04699123	II	Active, not recruiting
17	Myeloid immune checkpoint	TREM2	PY314 +/- pembrolizumab	Lung adenocarcinoma and other solid tumors	NCT04691375	I	Terminated
18	Myeloid immune checkpoint	ILT4/LILRB2	MK-4830 +/- pembrolizumab/chemotherapy	Dedicated NSCLC treatment arms	NCT03564691	I	Completed
19	Myeloid immune checkpoint	LILRB2	JTX-8064 +/- pimivalimab	NSCLC expansion cohort	NCT04669899	I/II	Completed
20	Myeloid immune checkpoint	ILT2/ILT4	NGM707 +/- pembrolizumab	Squamous and non-squamous NSCLC expansion cohorts	NCT04913337	I/II	Unknown
21	Myeloid immune checkpoint	PD-1 x ILT4	CDX-585	NSCLC and other advanced malignancies	NCT05788484	I	Completed
22	Myeloid immune checkpoint	LILRB2	OR502 +/- cemiplimab	NSCLC and other solid tumors	NCT06090266	I/II	Recruiting
23	Myeloid immune checkpoint	CD163	OR2805 +/- cemiplimab/docetaxel	NSCLC expansion cohort	NCT05094804	I/II	Unknown
24	Phagocytosis checkpoint	PD-L1 x CD47	PF-07257876	NSCLC, HNSCC, and ovarian cancer	NCT04881045	I	Completed
25	Phagocytosis checkpoint	PD-L1 x CD47	IBC0966	Phase IIa driver-negative NSCLC cohort	NCT04980690	I/II	Unknown
26	Phagocytosis checkpoint	Mesothelin x CD47	NI-1801 +/- pembrolizumab/paclitaxel	Mesothelin-positive non-squamous NSCLC	NCT05403554	I	Recruiting
27	Phagocytosis checkpoint	DLL3 x CD47	peluntamig (PT217) +/- standard therapy	SCLC/LCNEC and other DLL3-positive neuroendocrine carcinomas	NCT05652686	I/II	Recruiting
28	Phagocytosis checkpoint	PD-L1 x CD47	IMM2520	NSCLC/SCLC and other solid tumors	NCT05780307	I	Unknown
29	Phagocytosis checkpoint	HER2 x CD47	IMM2902	Advanced HER2-expressing lung cancer and other tumors	NCT05805956	I/II	Unknown
30	Engineered macrophages	HER2 CAR-M	CT-0508 +/- pembrolizumab	HER2-overexpressing solid tumors; registry includes NSCLC/SCLC	NCT04660929 (57)	I	Unknown

Unknown means that the registry record has not been verified within the required timeframe; it does not mean that the trial was terminated.

## 4 Discussion

### 4.1 Summary of basic information

Our analysis revealed several key patterns in lung cancer and TAM research. Regarding publication trends, the field has gone through three phases over the past 20 years, with a particularly significant acceleration in the last 7–8 years. Journal analysis indicates that papers are primarily published in oncology, immunology, and interdisciplinary journals, showing a vibrant flow of knowledge between basic and clinical research. At the national level, China leads in publication volume, but the US has a higher citation impact, and the two countries have the strongest collaborative relationship. At the institutional level, Chinese institutions occupy 9 of the top 10 positions in terms of paper publication, while US institutions such as MD Anderson Cancer Center have significantly higher average citations per paper. Collaborative networks among authors are relatively dispersed, with close ties within teams but weaker ties between teams. Keyword clustering analysis categorized existing literature into eight thematic groups, covering a wide range of topics from 2005 to 2018 and beyond, from chemotherapy resistance and metabolic reprogramming to immunotherapy.

Below, we will discuss the evolution of research themes, the current state and challenges of clinical applications, the main challenges in this field, and future prospects.

### 4.2 How research themes have evolved over two decades

Our results show that research on TAMs in lung cancer did not grow at a steady pace over the past two decades. From 2005 to 2015, annual publications increased slowly from fewer than 100 to about 150, and the research during this period was relatively fragmented. Publication growth picked up noticeably around 2018, and by 2025 the annual count had reached 439. That timing roughly coincides with the broad adoption of immunotherapy for lung cancer, suggesting the two are linked.

The keyword clusters provide a useful timeline of how the research focus shifted. Because our search scope is from 2005 to 2025, the earliest publications in this study can be traced back to around 2005, mainly focusing on nuclear receptors and DNA replication (58). In fact, this research direction belongs to an earlier era and has not further developed and grown in the future. A few years later, researchers turned to genetic polymorphisms in metabolic detoxification genes like GSTM1, GSTT1, and CYP1A1 (59–61). The prevailing hypothesis was that host genetic background affects lung cancer susceptibility. TAMs were not the main focus at the time. These genetic studies have continued since then, but they are no longer a central theme.

In the early 2010s, the field shifted toward chemotherapy. Studies around 2010 centered on drugs such as gemcitabine, cisplatin, and carboplatin (62–64). They asked two related questions: how do these agents change TAM phenotype and function, and conversely, how do TAMs contribute to chemoresistance? Of note, gemcitabine also appears in a separate cluster that emerged a few years later. That later cluster, with a mean year of 2012, focused more on TAM polarization, proliferation, and immunosuppressive functions (65–67).

After 2015, metabolic reprogramming entered the picture. Work from that period brought metabolism into focus, along with lung cancer invasion, metastasis and apoptosis (68–72). Cancer cells rely on aerobic glycolysis to fuel their growth, and TAMs also depend on specific metabolic pathways. However, the research in this area remains scattered, and no consensus has been reached on exactly how TAMs and tumor metabolism are linked. The relatively low silhouette score (0.348) of the metabolic reprogramming cluster indicates poor internal consistency. Rather than a mere lack of consensus, this structural fragmentation suggests fundamental methodological barriers in studying distinct metabolic subsets *in vivo*, or a conceptual divergence where researchers are struggling to uncouple TAM-specific metabolic traits from the broader tumor metabolic sink.

The most recent major shift occurred around 2018, with the rise of studies on lung adenocarcinoma, immune infiltration and pembrolizumab. Lung adenocarcinoma is the most common subtype of non-small cell lung cancer (73, 74). The timing of this cluster coincides with the time a few years after the approval of PD-1/PD-L1 antibodies for lung cancer (75–77). This suggests that basic research has been responsive to clinical needs. At the same time, we notice that terms related to small cell lung cancer do not appear in any major cluster, indicating that research on TAMs in SCLC is still at a very early stage. Only a very small number of studies have focused on this direction (78–80). The severe neglect of SCLC in TAM research is not coincidental; it is largely driven by the scarcity of matched surgical tumor tissues (as SCLC is rarely treated surgically), the aggressive nature of the disease leading to rapid deterioration, and the historical lack of robust immunocompetent murine models that accurately recapitulate the SCLC microenvironment.

Keyword burst detection tells a similar story. Early bursts were related to clinical pathological features such as staging and metastasis. In the later period, mechanistic related terms such as NF-κB, TGF-β and immunosuppression had emerged. Recent bursts point to pembrolizumab, macrophage polarization and combination therapy. That trajectory moves from describing phenomena to investigating mechanisms and then to translational work. Our results show keywords like stem cells, blockers, TME, lung adenocarcinoma, and macrophage polarization have gained attention in recent years (81–84). Current research focuses heavily on how immunotherapy affects TAM polarization and what role TAMs play in biological or immunotherapies. Our keyword analysis further shows that current research centers on chemotherapy and TAM-mediated resistance, factors affecting lung cancer progression and genomic biomarkers (85–88).

### 4.3 Key bottlenecks in current research

The publication output has grown quickly, but that doesn’t mean everything is moving smoothly. A few bottlenecks are holding the field back. One issue stands out as perhaps the most fundamental: the field still leans heavily on the M1/M2 polarization paradigm. Single-cell and spatial transcriptomic studies have repeatedly demonstrated that this binary classification is far too simplistic. In lung cancer, TAMs exhibit a continuum of activation states, and many cells co-express markers of both M1 and M2 (89). Moreover, the same TAM subset can display opposing functions depending on its location within the tumor (90, 91). The field has not yet established a unified standard for defining and naming TAM subsets. In most situation, we use CD86 and CD206 as prototypical M1/M2 markers, while include additional molecules like CD163, TREM2, or SPP1 (92–95). This lack of standardization makes cross-study comparison and meta-analysis extremely difficult, and it directly hampers the development of clinically useful biomarkers. For example, a recent study of CD163+ TAM density in non-small cell lung cancer showed high heterogeneity of the density, spatial distribution, and gene expression (96). Although there are some prognostic studies based on TAM subset levels, the prognostic value of TAM subgroups remains uncertain until consensus is reached on core biomarkers and reproducible scoring systems for TAMs (97–99).

From a biological perspective, the sheer heterogeneity of TAMs is a major challenge. Beyond the broad M1/M2 distinction, single-cell sequencing has uncovered some functionally distinct TAM subsets in lung cancer. The SPP1+ TAM subset is enriched in fibrotic regions and inhibits immune infiltration, reducing the immunotherapy efficacy (18, 100). Some researchers found the FOLR2+ TAM subset is near tertiary lymphoid structures and may retain some antigen-presenting capacity (101). But its role in tumor immunity still unclear. Then there’s the C1QC+TAM subset, which looks like it’s involved in complement activation and cleaning up cell debris, possibly creating an immunosuppressive mess (24, 102, 103). Different labs use different marker combinations and give these subsets different names. That’s a recipe for confusion. And as we discover more markers, it’s only going to get worse. We really need a standardized naming system and functional classification for human lung cancer TAMs subsets. Without such a framework, it will be very difficult to design drugs that selectively target tumor-promoting subsets while sparing tumor-inhibiting subsets.

Another bottleneck is the gap between mouse models and human patients. Subcutaneous xenografts can’t capture what TAMs really look like or how they’re organized in a real lung cancer patient. These preclinical models are limited exposure the human TME. So it’s common that many promising mouse targets flop in early human trials. CSF1R inhibitors is an example. In mouse lung cancer models, CSF1R inhibitor depleted TAMs and synergized with chemotherapy or immunotherapy (104–106). But in a clinical trial, the CSF1R inhibitor LY3022855 combined with durvalumab or tremelimumab in patients with advanced NSCLC had limited clinical benefits (107). Humanized mouse models and patient-derived xenografts are better, but they’re expensive and slow, not suitable for screening hundreds of compounds. And a lot of preclinical work still uses immunodeficient mice that have no T cells, B cells, or NK cells. We can’t study how TAMs interact with the adaptive immune system in those animals. This limitation may overestimate the efficacy of TAM-targeting agents when used alone and underestimates the importance of combination strategies.

We’d like to mention another key point. Most past studies have focused on NSCLC. SCLC gets almost no attention. That’s a problem because SCLC is more aggressive, spreads faster, and has few treatment options (108). Our keyword clusters contain almost no SCLC-specific terms, and a separate search confirms that fewer than 10% of published TAM-related lung cancer papers focus on SCLC. That’s a real gap. SCLC has its own distinct TME, with lots of immunosuppressive macrophages (109). There’s some early evidence that targeting the CD47-SIRPα axis might work in SCLC models, but solid clinical trial data are lacking (110).

Last bottleneck relates to the fragmentation of emerging themes and the imbalance between basic research and clinical application. Taking metabolic reprogramming of TAMs as an example, although it has attracted attention, the work in this field is still very scattered. The PKM2 cluster has a low silhouette value, meaning no coherent framework has emerged yet to connect TAM metabolism to whether they help or hurt the tumor. Some papers say lactate from tumor cells pushes TAMs toward an M2-like state through histone lactylation. Others say lactate actually helps macrophages suppress T cells under certain conditions (111–113). We don’t have standardized metabolic assays or good *in vivo* models to study TAM metabolism in a realistic setting.

### 4.4 What the findings mean for clinical translation and patient care

Basic research in this field has identified many potential targets, but only a few have moved into clinical trials and changed clinical practice. Among the highly cited references, one paper stands out: the work by Gordon and colleagues showing that PD 1 expression on TAMs inhibits phagocytosis, and that blocking PD 1/PD L1 restores macrophage phagocytic function (33). This finding directly linked TAMs to immune checkpoints and provided a rationale for combination therapy. TAMs can directly suppress T cells through PD L1 expression and also create a physical barrier by secreting chemokine. Combining anti PD 1 antibodies with TAM reprogramming agents is theoretically synergistic. Preclinical studies support this idea, but clinical studies are still limited. This gap is not unusual; it often takes more than ten years for a new target to move from discovery to validation and then to clinical implementation.

Although small in size, the cluster involving sarcopenia has clear clinical value. If TAM-related blood biomarkers could predict the risk of sarcopenia, or if modulating TAMs could improve patients’ functional status, which would directly benefit the quality of life of advanced lung cancer patients. Current evidence in this area is still fragmentary and has not formed a systematic research framework.

These studies aim to improve treatment outcomes, promote personalized treatment and enhance prognosis assessment. Based on our analysis of institutions, countries, highly cited references and recently used keywords, we believe future research on TAMs in lung cancer will move toward multi-omics integration to explore the spatial distribution and role of TAMs in lung cancer. For example, one study used single-cell sequencing to reveal the heterogeneity of tumor-associated macrophages in non-small cell lung cancer and differences between sexes; spatial transcriptomics has helped identify determinants of sensitivity and resistance to anti-PD1/PD-L1 antibodies related to macrophage infiltration (24, 114). At the same time, finding therapeutic targets on macrophages is an active area, for example macrophage CD5L is a target for cancer immunotherapy (115). Moreover, using machine learning and artificial intelligence for multi-omics integration is also becoming a new trend (116).

### 4.5 Promising directions for future research on TAMs in lung cancer

Based on the above discussion, we would like to highlight several directions that deserve attention. At the technological level, spatial transcriptomics and multiplex immunofluorescence are changing how we understand TAMs. Traditional methods lose spatial location information, whereas spatial transcriptomics allow us to see which TAM subsets are really close to tumor cells, which form immune synapses with CD8+ T cells, and which are kept behind fibrotic areas (117, 118). Preliminary applications have shown that SLC2A1+ TAMs as drivers of spatial CD8^+^ T cell exclusion, correlates with resistance to immunotherapy (118). In the future, integrating spatial data with single-cell transcriptomics to build a spatial map of TAMs in lung cancer may identify clinically meaningful targets.

Mechanistically, people are paying more attention to how TAM-derived chemokines shape immune infiltration in lung cancer, but a lot of it is still guesswork. We already know that macrophages in general can produce a range of chemokines, for example CCL2, CCL5, CXCL9, and CXCL10. In lung cancer, several studies have found that TAMs express higher levels of CCL2 and CCL5 than normal tissue macrophages (119–121). These chemokines are known to recruit monocytes, regulatory T cells, and myeloid derived suppressor cells. It is therefore plausible that TAM derived CCL2 and CCL5 contribute to the accumulation of immunosuppressive cells within the TME (122, 123).

Several unresolved questions make it difficult to draw firm conclusions. It is not known whether specific TAM subsets, such as SPP1+ or FOLR2+ macrophages, have distinct chemokine secretion profiles, because most existing studies rely on bulk tumor lysates or *in vitro* differentiated macrophages that do not capture subset specific heterogeneity. Spatial transcriptomic data are beginning to map chemokine gradients, but these studies are correlative and cannot establish causality. A more definitive understanding will likely require genetic approaches in animal models combined with well-designed human correlative studies that account for TAM subset identity and spatial location.

For clinical application, we believe the most urgent need is to establish standardized protocols for TAM detection and subset classification. Retrospective studies have shown that CXCL9/SPP1 TAM correlates with prognosis, but staining procedures, antibody lots and scoring criteria are not uniform, so these results cannot be directly used in clinical practice (124). We could learn from the standardization of Ki67 and PD-L1 in breast cancer, and have professional thoracic pathology groups develop guidelines for TAM immunohistochemistry scoring in lung cancer. For more complex subset classification, multiplex fluorescence immunohistochemistry panels with automated image analysis software may be needed.

Regarding therapeutic strategies, dual-target or multi-target agents may be more promising than single targets. For example, specific antibodies against CSF1R could theoretically reduce TAM numbers while relieving T cell suppression (125, 126). Because PD-L1 is highly expressed mainly in the TME, such targeting might reduce off-target killing of normal tissue macrophages. CAR-macrophages are another new direction, with initial safety validation already completed in a few models of solid tumors (127–129). However, how to choose suitable lung cancer associated antigens, how to control the persistence of CAR-M, and how to avoid cytokine storms are issues that need to be addressed one by one. Our analysis shows that publications on CAR-M have grown rapidly in the last two years, but most are still at the proof-of-concept stage. The “CAR-M” field was retrieved in WOSCC, and there were 7 articles in 2023, 9 articles in 2024, and 12 articles in 2025. This search is a supplementary search, not a core clustering analysis. Although CAR-M does not emerge as a high-frequency citation burst or standalone cluster due to its very recent inception, its exponential publication growth in general oncology highlights its rising momentum, necessitating its inclusion as an emerging frontier. The next decade will be critical to determine whether this technology can go further in lung cancer.

We want to mention a relatively neglected area: small cell lung cancer. SCLC does not appear in any major cluster in our analysis, indicating that research is still very limited. Yet SCLC is more aggressive, has fewer treatment options, and its TME may have unique features. If specific TAM-related targets could be identified in this subtype, the clinical value would be high. Similarly, common comorbidities in lung cancer patients, such as sarcopenia, cachexia and chronic obstructive pulmonary disease, and their interactions with TAMs, deserve more attention. Although these directions currently have low publication volumes, they may well be future growth points. Based on our analysis of institutions, countries, highly cited references and recently used keywords, we believe future research on TAMs in lung cancer will move toward multi-omics integration to explore the spatial distribution and role of TAMs, to investigate in detail how to enhance biological or immunotherapy for lung cancer (stem cell vaccines, targeted drugs, macrophage polarization), to find therapeutic targets on macrophages, and to develop personalized treatment strategies using machine learning and artificial intelligence (130).

### 4.6 Strengths and limitations of this study

We need to acknowledge both strengths and limitations as a bibliometric study. To obtain a comprehensive view of the literature on lung cancer and TAMs, we used the WOSCC and Scopus database for its broad, reliable and unbiased coverage, and then performed bibliometric analysis and visualization with VOSviewer and CiteSpace. This approach facilitates the analysis of current research hotspots and future trends. Our study demonstrates the advantages of bibliometrics in citation analysis, identification of high-impact publications, interdisciplinary research and data quality, enabling in-depth literature analysis and tracking of academic development.

However, this work has several limitations. We could not directly access the raw data from the publications and had to rely entirely on information extracted by the analysis tools. Although both databases aim to cover all disciplines, it underrepresents the social sciences and humanities. Furthermore, both WOSCC and Scopus suffer from inherent structural limitations, notably the systematic under-representation of regional non-English biomedical literature and inevitable citation lags that disadvantage recently published breakthrough articles. Restricting the language to English may introduce language bias. In addition, differences in the attribution of authors and institutions may lead to biased statistical results. One more thing to keep in mind: our search was done at a specific time, with a specific formula, and databases don’t update instantly. So the data we analyzed probably misses some recent papers. Our interpretation of publication counts, citation frequencies and keywords may carry some subjectivity.

## 5 Conclusion

We believe lung cancer and TAMs research has moved from M1/M2 descriptions to heterogeneity, metabolism, and chemokine regulation. In our view, immunotherapy combinations and spatial technologies are current frontiers, but most therapeutic approaches remain preclinical with unresolved safety issues. We are concerned by the lack of standardized TAM subset nomenclature, limited clinical validation, and neglect of small cell lung cancer. By establishing a consensus framework among peers and conducting targeted cohort studies, these bottlenecks can be overcome. We call for more cross-institutional collaboration and rigorous testing to translate lab discoveries into patient benefit.

## Data Availability

The original contributions presented in the study are included in the article/**Supplementary Material**. Further inquiries can be directed to the corresponding author.

## Supplementary material

Supplementary Figure 1 Funding landscape for lung cancer TAM research.

Supplementary Figure 2 Citation burst analysis of key references in lung cancer TAM research.
